# Online Questionnaire with Fibromyalgia Patients Reveals Correlations among Type of Pain, Psychological Alterations, and Effectiveness of Non-Pharmacological Therapies

**DOI:** 10.3390/healthcare10101975

**Published:** 2022-10-09

**Authors:** Ilaria Demori, Elena Molinari, Fabio Rapallo, Viviana Mucci, Lucio Marinelli, Serena Losacco, Bruno Burlando

**Affiliations:** 1 Department of Earth, Environmental and Life Sciences (DISTAV), University of Genova, Corso Europa, 26, 16132 Genova, Italy; 2 Clinical Psychology Unit, E.O. Ospedali Galliera, Via Mura delle Cappuccine 14, 16128 Genova, Italy; 3 Department of Economics (DIEC), University of Genova, Via Vivaldi, 5, 16126 Genova, Italy; 4 School of Science, Western Sydney University, Penrith, NSW 2150, Australia or; 5 Department of Neuroscience, Rehabilitation, Ophthalmology, Genetics, Maternal and Child Health, DINOGMI, University of Genova, Largo P. Daneo 3, 16132 Genova, Italy; 6 IRCCS Ospedale Policlinico San Martino, Department of Neuroscience, Division of Clinical Neurophysiology, Largo R. Benzi 10, 16132 Genova, Italy; 7 Department of Pharmacy, DIFAR, University of Genova, Viale Benedetto XV 3, 16132 Genova, Italy

**Keywords:** non-pharmacological therapies, neuropathic pain, nociplastic pain, online survey, patient management, psycho-neuro-endocrine-immunology

## Abstract

Fibromyalgia (FM) is a chronic pain syndrome with an unclear etiology. In addition to pain, FM patients suffer from a diverse array of symptoms and comorbidities, encompassing fatigue, cognitive dysfunction, mood disorders, sleep deprivation, and dizziness. Due to the complexity of FM, the diagnosis and treatment of it are highly challenging. The aim of the present work was to investigate some clinical and psychological characteristics of FM patients, and to uncover possible correlations with pharmacological and non-pharmacological therapies. We conducted a cross-sectional, questionnaire-based study aimed at evaluating pain, psychological traits, and the self-perceived effectiveness of pharmacological and non-pharmacological treatments in an Italian population of FM patients. Descriptive statistics, correlation, and inference analyses were performed. We found a prevalence of a neuropathic/nociplastic type of pain, which correlated with psychological traits such as anxiety, low mood, psychophysical discomfort, and the inability to relax. The pain type and psychological traits proved to play a role in determining the self-perceived effectiveness of therapeutic interventions. Patients revealed a better response to non-pharmacological therapies, particularly dietary interventions, relaxation techniques, and psychotherapy rather than pharmacological interventions. The sum of our data indicates that for better outcomes, the type of pain and psychological traits should be considered for tailor-made treatments considering non-pharmacological protocols as a complement to the use of drugs.

## 1. Introduction

Fibromyalgia (FM) is a complex syndrome mainly characterized by multisite pain and moderate-to-severe sleep problems or fatigue [1,2]. The syndrome has a prevalence of about 2–5% in the adult population, mostly affecting women with a reportedly variable male-to-female ratio of around 1:9 [3]. Despite that FM was firstly described in 1904 [4], the causes of the disease remain unknown. Its primary site of localization, either peripheral or central, is debated, while many assume that the condition is multifactorial in origin [5]. However, the accumulated evidence suggests that FM is a central pain processing disorder that generates pain from non-painful or mildly painful stimuli [6]. FM patients suffer from allodynia and hyperalgesia, two common features of the central sensitization process of pain amplification in the central nervous system [7].

The lack of clear pathophysiology is correlated with the lack of biomarkers, making the diagnosis of FM often challenging. The latest updated diagnostic criteria formulated by the American College of Rheumatology have set threshold values for a series of parameters, including symptom duration, widespread pain index, and a symptom severity scale [8]. However, a long list of symptoms and comorbidities may be present in FM patients, including anxiety, headaches, irritable bowels, joint stiffness, mood disorders, paresthesia, sleep disorders, and dysautonomia. As a result, not only patient diagnosis but also patient management are complex and difficult, thus hindering the introduction of standardized therapeutic guidelines [9,10,11,12]. Either pharmacological or non-pharmacological treatments can be adopted, with medications including anti-seizure drugs, anti-inflammatories, antidepressants, analgesics, and muscle relaxers, while most common non-pharmacological and alternative treatments include physical therapy, massages, relaxation, psychotherapy, acupuncture, and diet therapy [13]. A variable outcome for such a diverse panel of treatments has been reported in a series of clinical studies and surveys, and despite some effectiveness of both strategies has been observed, no resolutive approach has yet been identified and no clear comparison between the pharmacological versus the more holistic or non-pharmacological approaches has ever been made [14,15,16].

Considering the drawbacks in FM diagnosis and patient management, there is a clear need to formulate a tenable pathophysiological model able to explain the insurgence of the disease. In this regard, the epidemiological traits and comorbidities associated with FM can provide useful insights into its etiology. The marked female-biased sex ratio and perimenopausal prevalence suggest a gonadal involvement [17], while several comorbidities are also female-prevalent, such as depression [18], migraines [19], and central vestibular disorders [20]. In addition to the hormonal aspects, correlations with early childhood adversities and psychosocial stress in FM patients have been considered. Stress is known to impair the hypothalamic-pituitary-adrenal axis (HPA) with cascade repercussions on neurosteroid metabolism [21], which could explain the chronic fatigue associated with FM, and on the immune system [22], which can also interfere with pain processing [23]. Moreover, the possible role of immune responses in FM pathogenesis has been consistently considered [24,25].

FM correlations with immunoendocrine and psychological factors come together with the recognized importance of body-mind aspects in the management of the disease [26]. Psycho-Neuro-Endocrine-Immunology (PNEI) is a new discipline that integrates scientific knowledge from both psychological and biological sciences and describes the complex relationships between psychosocial processes and the nervous, endocrine, and immune systems, thus highlighting the bidirectional connections between body and mind, and proposing a systemic multidimensional approach to human health [27,28,29]. In this scenario, we aimed to explore, through questionnaires, the clinical characteristics of FM patients in Italy, by asking which types of therapies they considered most effective, and by exploring the correlations between pain type, psychological traits, and treatment effectiveness.

## 2. Materials and Methods

### 2.1. Participant Recruitment

An online anonymous survey was opened from April to June 2021, accessible through a link on the Microsoft Office365 Platform of the University of Genova (Microsoft Forms^®^, https://www.office.com (accessed on 22 February 2021), Microsoft, Redmond WA, USA). Participants were recruited thanks to the collaboration of an Italian association of FM patients (Fibromialgia Comitato Assoutenti Liguria, http://fibromialgiaediritti.altervista.org (accessed on 8 March 2021)), who shared with 520 addresses from its mailing list an information letter and the link to the survey. Participants joined the survey individually, freely, and anonymously. The acceptance of a full informed consent was mandatory to be able to start the survey. Patients aged between 18 and 65 years with an established FM diagnosis were included in the study (symptoms having been present for at least 3 months [8]). Exclusion criteria were the following: not being able to understand and write the Italian language, pregnancy, breastfeeding, substance and alcohol abuse, diagnosis of psychiatric comorbidities included in the spectrum of schizophrenia, and other psychotic disorders. The study was approved by the University of Genova Research Ethics Committee (Assent N. 2021/32).

### 2.2. Questionnaires

The first section of the survey assessed socio-demographic characteristics such as age, gender, family status, educational status, and working status, as well as clinical aspects including biometric parameters, age-of-onset, diagnostic delay, and disease duration.

For pain evaluation, participants reported their average global pain intensity over the past week on an 11-point Numerical Rating Scale (ranging from 0 = “no pain” to 10 = “worst imaginable pain”). A more detailed analysis of pain was realized through a survey of a series of pain types, including pressure pain, numbness, tingling, sudden pain, burning, light contact, and occasional pain, on a scale from 1 to 5. Finally, participants answered the Italian translation of the painDETECT questionnaire (PD-Q), developed to detect neuropathic pain components, especially in chronic patients [30]. A PD-Q score ≤ 12 indicates that a neuropathic pain component is unlikely, a PD-Q score ≥ 19 indicates that a neuropathic or central pain component is >90% likely, and an intermediate condition is considered in between.

A study-specific form was designed to investigate the self-perceived effectiveness (null, low, average, good, and excellent) of pharmacological treatments (analgesics, antidepressants, non-steroidal anti-inflammatory drugs, and steroids) and non-pharmacological ones (acupuncture, diet therapy, massage therapy, non-invasive instrumental treatments, physical therapy, psychotherapy, and relaxation therapy).

In the third section of the survey, participants answered well-validated Italian versions of two questionnaires: the Cognitive Behavioural Assessment-Hospital (CBA-H) [31], and the Self-Rated Emotional Intelligence Scale (SREIS) [32]. The CBA-H questionnaire consists of broad-spectrum “true/false” questions organized into 3 parts (A, B, C), aiming at multiple evaluations including anxiety, well-being, depression, psychological distress, fear, and stable personality traits. The SREIS test investigates abilities related to Emotional Intelligence, such as perceiving, using, understanding, and managing emotions. Participants answered on a 5-point Likert scale (ranging from 1 = “not at all” to 5 = “very much”), indicating how accurately each item describes their psychological profile.

### 2.3. Statistical Analyses

Data of sociodemographic and clinical characterizations were used for descriptive statistics. Correlation and inference analyses were applied to the following variables: intensities of seven types of pain (light contact, occasional pain, burning, tingling, sudden pain, numbness, pressure pain), PD-Q scores, perceived effectiveness of treatments, and CBA-H and SREIS scores. Where appropriate, the goodness of fit for categorical variables was assessed by means of the chi-square test, while the difference in distribution for semi-quantitative scores was assessed by means of the Mann–Whitney test. For a multivariate analysis, the clustering of the variables was performed with the average linkage agglomerative algorithm based on pairwise correlations. The questionnaire scale reliability was evaluated for internal consistency according to Cronbach’s alpha (0–1.0). Data analyses were carried out using the software R (version 4.0.5, https://www.r-project.org/ (accessed on 5 July 2021)) and MatLab (R2021, MathWorks, Natick, MS, USA).

## 3. Results

### 3.1. Sociodemographic Characteristics

The survey received 352 answers, i.e., 67.69% of the total number of invitations sent to FM patients. Among the participants who specified their gender (n = 324), about 88% were females. Respondents were on average 47.9 years old (median = 50). A large proportion of the enrolled patients had a high level of education (about 81% upper secondary/academic degree, PhD, or equivalent), while most of them were married or cohabitant with a partner (about 60%). Half of the patients had children, and jobs as white-collar or grey-collar workers (about 50%) (Table 1).

### 3.2. Clinical Characteristics

The biometric data reported by participants allowed for the derivation of body mass index (BMI) values (Table 2), showing that the prevalence of obesity (BMI > 30) was about 20%. Statistics concerning the age of onset and diagnostic delay were typical of the disease.

**Figure 1:**
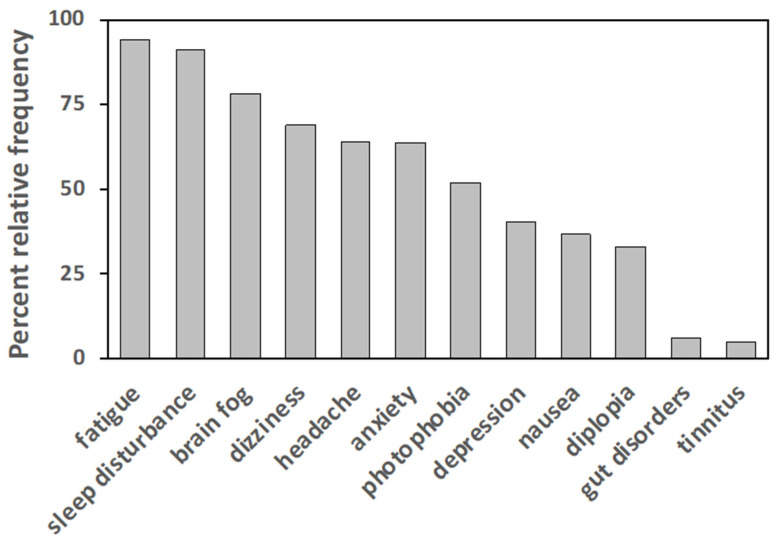
Percent relative frequencies of symptoms reported by participants aside from pain. For each symptom, the value represents the percentage of patients reporting that symptom (n = 352)**.** Frequencies lower than 4% have been omitted.

Participants were also asked to report symptoms other than pain. Typically, FM-associated symptoms or comorbidities, such as fatigue, sleep disturbance, brain fog, dizziness, headache, anxiety, photophobia, depression, gastro-intestinal disorders, and diplopia, have been reported with different relative frequencies, as shown in Figure 1.

### 3.3. Psychological Profile

**Figure 2:**
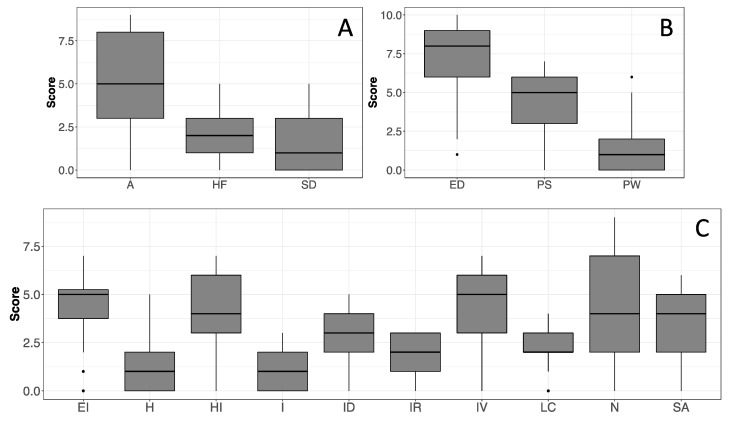
Boxplots of the scores obtained in each scale of the CBA-H. Scale legends with Cronbach’s alpha values in parentheses; (**A**) A = State anxiety (0.87); HF = Health-care related fears (0.54); SD = Situational depression (0.67); (**B**) ED = Depressive mood (0.71); PS = Perceived psychophysical stress (0.68); PW = Psychophysical wellbeing (0.57); (**C**) EI = Excessive involvement (0.42); H = Hostility (0.50); HI = Haste and impatience (0.59); I = Irritability (0.32); ID = Interpersonal difficulties (0.64); IR = Inability to relax (0.64); IV = Introversion (0.76); LC = Leadership/Competitiveness (0.10); N = Neuroticism (0.79); SA = Social anxiety (0.73). Sample size, n = 352.

**Figure 3:**
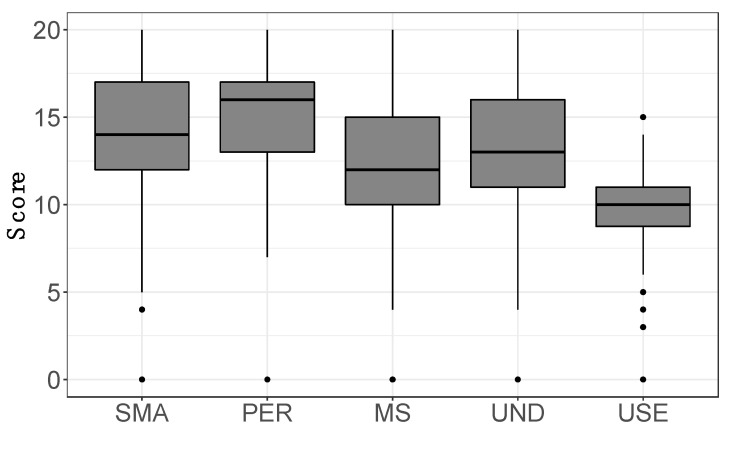
Boxplot of the score distributions for the 5 scales of the SREIS test. Scale legend: SMA = Social management of emotions; PER = Perceiving emotions; MS = Managing emotions “self”; UND = Understanding emotions; USE = Use of emotions. Sample size n = 352.

Regarding psychological characteristics, according to the clinical cutoffs of the CBA-H (Cronbach’s alpha in Figure 2), the study population presented subclinical state anxiety (Figure 2A), low mood (Figure 2B), and emotional over-involvement (Figure 2C) in the last 3 months, leading to psycho-physical discomfort without signs of psycho-pathological behavior. The SREIS scores (Cronbach’s alpha = 0.84) revealed that participants tend to have a high ability to perceive emotions, but a lower capacity to understand and self-manage them (Figure 3), possibly leading to critical emotional stability.

### 3.4. Evaluation of Pain

**Figure 4:**
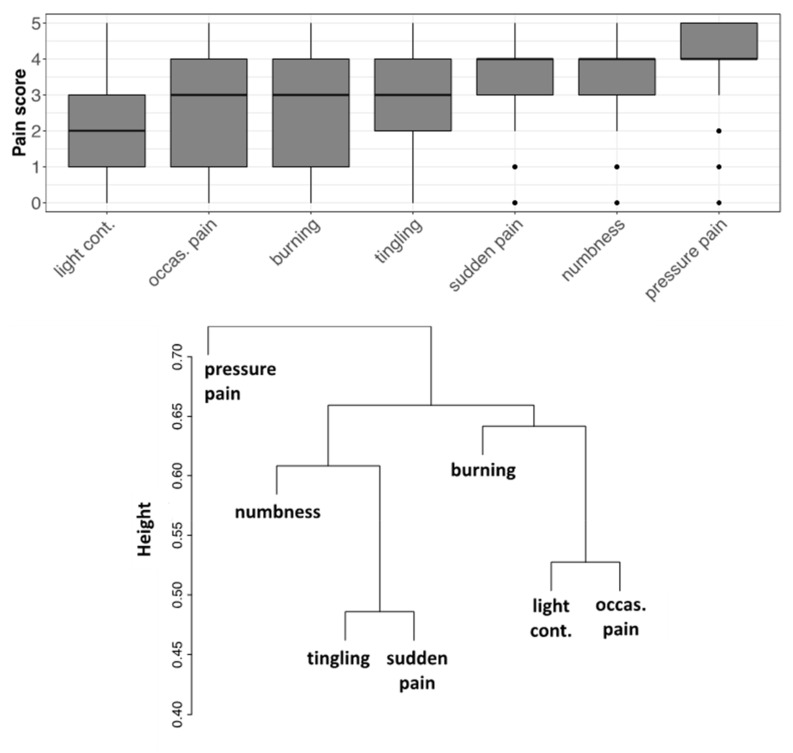
Scores reported by subjects for each pain type. (**Top**) Boxplots of score distributions for each type of pain experienced by patients in the last week before questionnaire compilation. (**Bottom**) Dendrogram of the different types of pain, generated using an average linkage agglomerative algorithm based on pairwise correlations between pain intensities. The distance used is one minus the correlation coefficient. The height of each node (vertical axis) is the distance value between the right and left sub-branch clusters. Light cont. = light contact; Occas. pain = occasional pain; Sample size n = 352.

Participants indicated an average pain intensity level of 6.4 ± 1.8, on a scale from 0 to 10, referring to the last week prior to the questionnaire. A fraction of 76% of patients experienced a pain level above 6, which indicates moderate-to-severe pain. The survey of the different types of pain showed the highest scores for pressure pain, while a dendrogram analysis of the correlations among pain types showed that pressure pain was the most uncorrelated from any other one, whereas tingling and sudden pain, and light contact and occasional pain formed two strictly correlated clusters, respectively (Figure 4).

**Figure 5:**
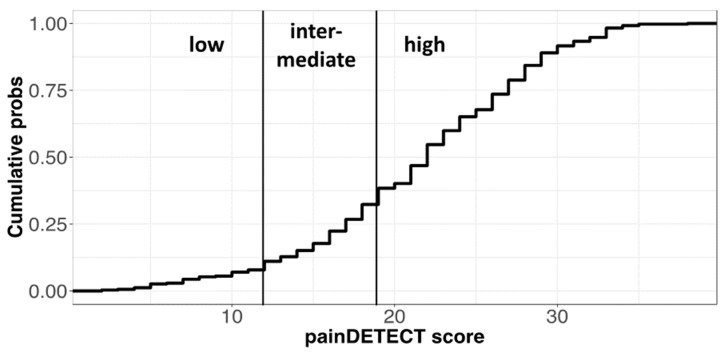
Scores of the PD-Q test and related categories. (Top) Cumulative frequency of scores in the PD-Q scale with cutoffs for pain categories. The relative frequencies of pain categories in the test (low = 0.11; intermediate = 0.21; high = 0.68) are significantly different from uniform distribution according to a chi-square test (n = 344, χ-squared = 188.5, df = 2, *p* < 2.2 × 10^−16^).

The PD-Q test (Cronbach’s alpha = 0.78) resulted in a prevalence of high scores, and consequently, a significantly unequal distribution of patients among the three pain categories defined by the test score cutoffs, with a marked prevalence of “high” subjects with respect to “low” and “intermediate” ones (Figure 5). “High” subjects are considered to have a distinct component of neuropathic pain, which, according to the definition followed by PD-Q developers, might also correspond to central pain processing [30], i.e., being compatible with the more recent notion of nociplastic pain used to define pain processing disorders [33].

**Figure 6:**
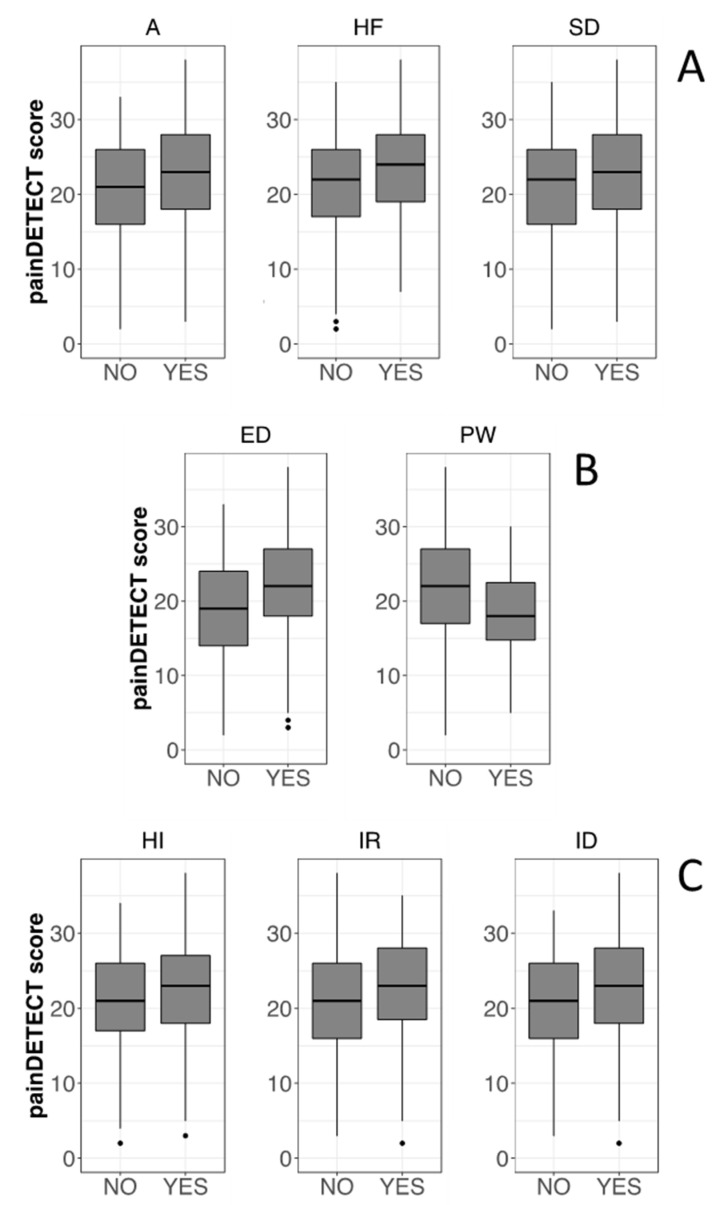
Boxplots showing the correlation between PD-Q scores and CBA-H categories. For each category of the CBA-H test, two groups have been identified based on the cutoff for the presence of the related clinical concern, indicated by the YES label, except for PW (Psychophysical wellbeing) where YES indicates a positive condition. Boxplots have been then obtained showing the frequency distributions of the PD-Q scores for each of the two groups. Only the categories with significant differences between the NO and YES cases are shown (Mann–Whitney test, *p* < 0.05). CBA-H test; (**A**) (top): A = State anxiety; HF = Health-care related fears; SD = Situational depression. CBA-H; (**B**) (middle): ED = Depressive mood; PW = Psychophysical wellbeing. CBA-H; (**C**) (bottom): HI = Haste and impatience; IR = Inability to relax; ID = Interpersonal difficulties.

A correlation between the PD-Q test scores and the CBA-H clinical cutoffs has been investigated, and the significant results are reported in Figure 6. As an overall trend, the PD-Q score was higher when CBA-H cutoffs indicated a clinical concern in the corresponding category. Thus, significantly higher PD-Q scores have been detected in the presence of state anxiety, health-care-related fears, situational depression, haste and impatience, the inability to relax, and interpersonal difficulties. In the case of psychophysical wellbeing, higher PD-Q scores are detected when the condition was absent.

### 3.5. Treatment Effectiveness

**Figure 7:**
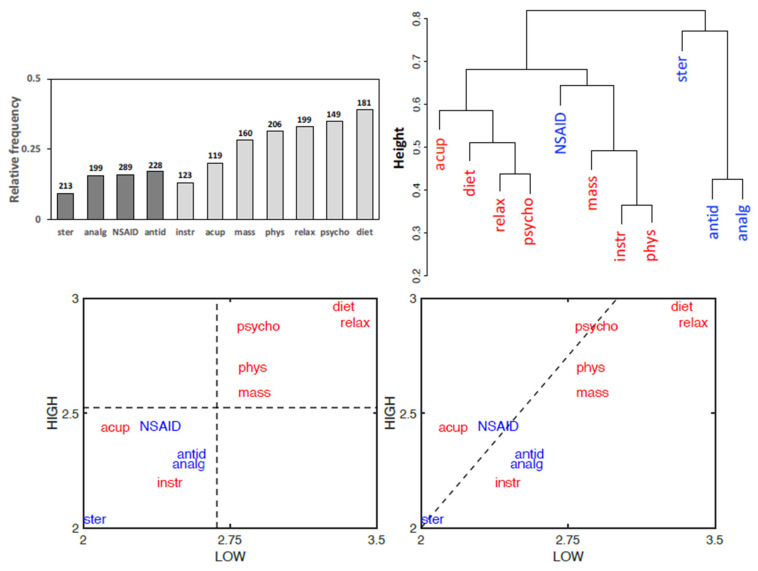
Patient’s classification of the therapy efficacies. (**Top left**) Cumulated relative frequencies of the two highest ratings out of five (null; low; average; good; excellent). Numbers above bars indicate total valid responses. Darker bars: pharmacological treatments; lighter bars: non-pharmacological treatments. (**Top right**) Dendrogram generated using an average linkage agglomerative algorithm based on pairwise correlations between the effectiveness of the different treatments (Pharmacological treatments = blue; non-pharmacological treatments = red; data as in Figure 4). (**Bottom**) Bidimensional plots of the pharmacological (blue) and non-pharmacological (red) treatment effectiveness. The coordinates of each treatment are the average values of effectiveness reported by subjects rating as “low” (horizontal axis) or “high” (vertical axis) in the PD-Q test. In the left panel, the perpendicular dotted lines, intersecting axes at global mean values, show that most non-pharmacological treatments are generally more effective than pharmacological ones. In the right panel, the axes bisector (dotted line) shows that most treatments are judged more effective by “low” subjects. Statistical comparisons by the Wilcoxon test show that non-pharmacological treatments are judged more effective than pharmacological ones by the whole population of patients (n = 344, *p* = 2.03 × 10^−11^), as well as by analyzing separately “high” subjects (n = 233, *p* = 5 × 10^−8^) or “low” subjects (n = 38, *p* = 0.018). Mind-body therapies have been reported as the most effective in absolute. Non-pharmacological treatments: acup = acupuncture; diet = diet therapy; mass = massages; instr = non-invasive instrumental treatments; phys = physical therapy; psycho = psychotherapy; relax = relaxation therapy. Pharmacological treatments: analg = analgesics; antid = antidepressants; NSAID = non-steroidal anti-inflammatory drugs; ster = steroids.

The perceived effectiveness of therapies reported by patients revealed in almost all cases a better response to non-pharmacological treatments with respect to pharmacological ones, as also highlighted by the clustering together of most non-pharmacological therapies in a dendrogram analysis (Figure 7). The categories of the PD-Q test have been used to further investigate the pattern of effectiveness of the different therapies. A plot of the average effectiveness reported for each pharmacological and non-pharmacological therapy by the “low” and “high” patients of the PD-Q test confirms higher effectiveness for non-pharmacological treatments, except acupuncture and instrumental physical therapy. The plot also shows that a higher effectiveness for most therapies is tendentially reported by “low” patients with respect to “high” patients, suggesting a negative correlation between therapy effectiveness and central nervous problems (Figure 7). A similar pattern is obtained if “intermediate” subjects are also considered in a three-dimensional plot (not shown).

**Figure 8:**
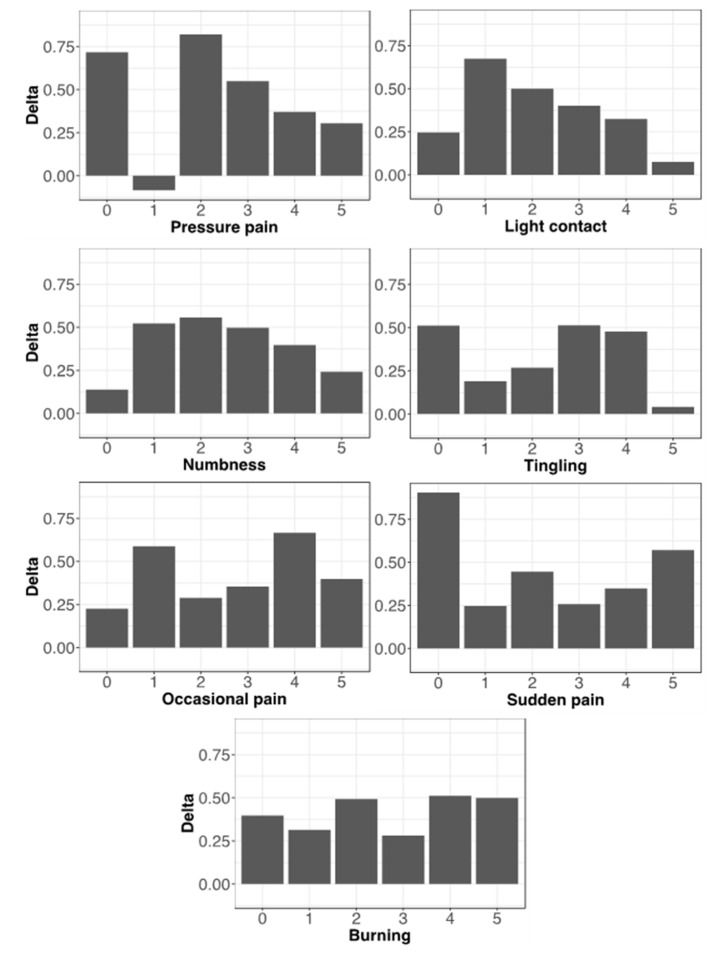
Correlations between treatment effectiveness and pain types. Bars (Delta values) represent differences between the average effectiveness of non-pharmacological and pharmacological therapies for each level of intensity of the different types of pain. Positive values indicate the prevalence of non-pharmacological treatments, which is almost total but tends to decrease with higher values of pain intensity in pressure pain, light contact, numbness, and also in tingling, though with a biphasic trend. The countertrend value at intensity level “1” of pressure pain could represent a random fluctuation due to the limited number of data (n = 7).

If the different types of pain are considered, each subdivided into intensity levels, the higher effectiveness of non-pharmacological treatments with respect to pharmacological ones is almost totally confirmed across all pain types, though in some of them the prevalence of non-pharmacological treatments tends to diminish with the increasing severity of pain (Figure 8).

**Figure 9:**
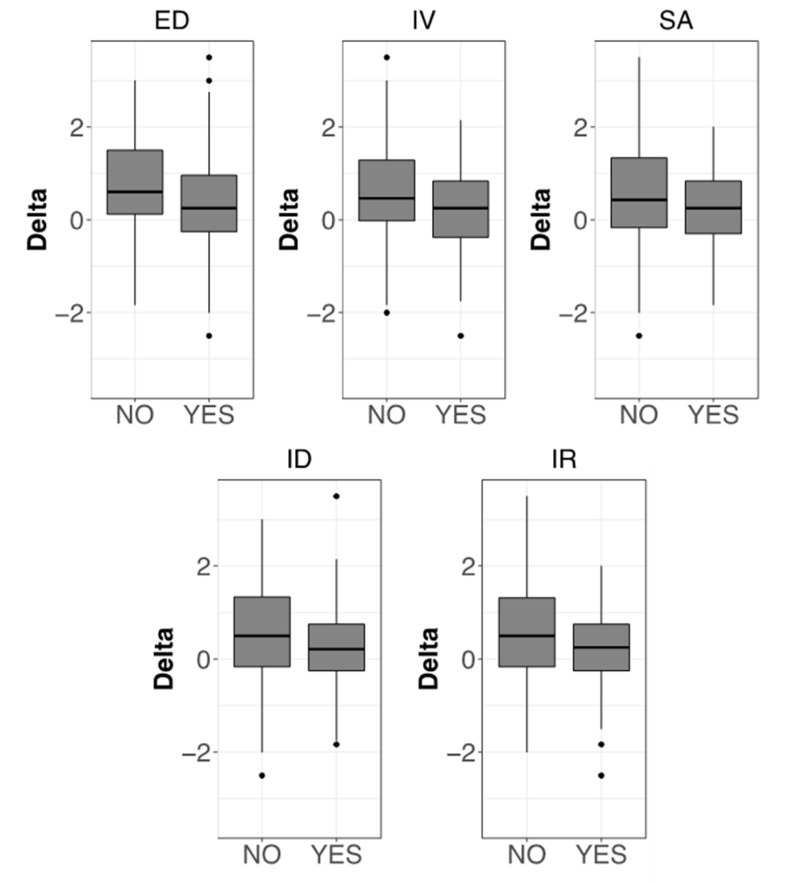
Boxplots showing the correlation between therapy effectiveness and different CBA-H test categories. For each category of the CBA-H test, two groups have been identified based on the cutoff for the presence of the related clinical concern (see Figure 6). Delta values (vertical axis) represent differences between the average efficacies of non-pharmacological and pharmacological therapies. Positive values indicate the prevalence of non-pharmacological treatments. Only the categories with significant differences between NO and YES cases are shown (Mann–Whitney test, *p* < 0.05). CBA-H part B (left top): ED = Depressive mood. CBA-H part C: IV = Introversion; SA = Social anxiety; ID = Interpersonal difficulties; IR = Inability to relax.

A correlation between the CBA-H clinical cutoffs and the perceived effectiveness of non-pharmacological and pharmacological therapies has also been investigated, and significant results are reported in Figure 9. Non-pharmacological therapies have been found relatively less effective by patients with emotional instability, introversion, social anxiety, interpersonal difficulties, and an inability to relax.

## 4. Discussion

### 4.1. FM Clinical Management Is Affected by Diagnostic Drawback and Delay

The sociodemographic and clinical characterizations of our sample of participants confirmed the typical FM patient profile: a high number of female patients, the onset of symptoms occurring at adult age prior to menopause (average age 42.3 ± 10.1 years), and a marked diagnostic delay [34,35]. Data about pain measurement and types of pain were fairly consistent with the typical clinical features of FM, particularly the occurrence of the highest scores for pain pressure, since a lower pain pressure threshold is considered a classic FM diagnostic element [36].

Symptoms reported in addition to pain are related to current diagnostic guidelines [2]. Sleep problems and fatigue are prevalent in our cohort, followed by perceptual disturbances, headaches, anxiety, and low mood. Shortcomings in the diagnostic process are also relevant: patients were subjected to a significant diagnostic delay (6.35 ± 6 years on average), confirming the difficulties encountered in the clinical characterization of FM. These drawbacks leave patients “in limbo”, uncertain about their future, and in a state of chronic stress, thus representing a major concern for the optimal management of the syndrome. According to the EULAR recommendations (European League Against Rheumatism), a prompt diagnosis is of the utmost importance and could allow gradual therapeutic approaches for a more comprehensive assessment considering pain, other symptoms or comorbidities, and the psychosocial context [37].

### 4.2. FM as a Central Multisensory Disorder

The pattern of perceived pain revealed the typical FM traits, with pressure pain being dominant and uncorrelated from other types of pain. The marked prevalence of this pain component is compatible with a central disorder of pain processing, according to the recent view of nociplastic pain [33]. Moreover, pain scores tended to be high, indicating severe pain for most participants. In this context, the CBA-H test gave some interesting results. First, it showed that participants experienced state anxiety, low mood, and emotional discomfort. These symptoms could be related to the experience of pain and particularly to “pain catastrophizing” (i.e., a maladaptive cognitive-emotional tendency to consider pain terrible and intolerable), which is common among FM patients [38]. Secondly, we found completely new correlations between psychological alterations and the types of pain. Our data fit the revised definition of pain delivered in 2020 by the International Association for the Study of Pain: “An unpleasant sensory and emotional experience associated with, or resembling that associated with, actual or potential tissue damage”. This definition is also expanded by key notes highlighting that pain is a personal experience influenced by physiological and psychosocial factors, and that individuals develop the concept of pain through their life experiences [39].

Although pain is generally perceived as the most annoying symptom of FM, many other symptoms or co-morbidities are present and often reported as debilitating and impairing by patients. Despite a variable spectrum and different frequencies among patients, symptoms such as chronic fatigue, sleep disturbance, brain fog, depression, anxiety, headaches, and an irritable colon often occur [40]. Our data confirmed this pattern, but it is worth noting that sensory symptoms like dizziness, vision disturbances, and in some cases, tinnitus, have also been reported. This suggests that the thalamic region plays a pivot role in FM insurgence, as the main relay station of sensory signals. This idea is strengthened by the remarkably similar arrangement of the thalamocortical networks involved in the pain and visual processing areas [41,42]. Other possible hints that sustain the supraspinal origin and localization of FM are the low effectiveness of analgesics (acting at the spinal level), of anti-inflammatory drugs (mostly acting peripherally), and of antidepressants (aimed at potentiating descending pain control pathways, from monoaminergic nuclei to the spinal cord) [43]. In addition to this, the supraspinal hypothesis could be further supported by the highest effectiveness reported for mind-body treatments, allegedly acting directly or indirectly on brain networks. Hence, our data suggest that FM should be considered as a central, multisensory disorder, rather than a purely chronic pain disorder, thus being in line with the hypothesis of “centralized sensitivity syndrome” [10], and emphasizing that this aspect deserves careful diagnostic and clinical inspection.

### 4.3. Non-Pharmacological Therapies Prevail over Pharmacological Ones

FM subjects are frequently treated by combined therapies consisting of standard medications and non-pharmacological therapies or alternative medicines [16]. In our sample, a lower effectiveness was reported for pharmacological therapies. This could reflect the chance of several side effects often occurring with such medications. On the other hand, non-pharmacological remedies, which received higher scores, are possibly able to ease the side effects of drugs. However, given the well-assessed mental component of the FM syndrome [44], and since the highest scores given in the questionnaire were to mind-body approaches like dietary interventions, relaxation, and psychotherapy, it is also possible that these treatments exert their action close to the core of the disease, whereas pharmacological strategies seem not able to adequately hit critical therapeutic targets. In any case, our results confirm the difficulties of prescribing suitable drugs to FM patients [45] and are in line with EULAR recommendations stating that the primary outcome of FM management should be improving the health-related quality of life, achieved through a multi-disciplinary approach balancing the benefits and the risks of treatments and proceeding gradually, starting from non-pharmacological treatments [37].

Given the clear benefits of non-pharmaceutical interventions and the psychosomatic component reported by our cohort, our results confirm the relevance of alternative treatments in FM patients and the importance of considering the psychological component of the disease [45,46,47]. Our findings highlight for the first time a correlation between psychological alterations and central pain in the same patients. These insights can be combined with the known involvement of chronic stress in FM [48], which was confirmed in our questionnaire by the presence of typical pain-associated symptoms such as sleep disturbance and fatigue, and with the known role of an immunoendocrine imbalance in FM pathogenesis. This view could improve our understanding of the etiological mechanisms, possibly in terms of a central pain processing disorder with multiple upstream causes, thereby leading to the development of more targeted therapeutic strategies. Consistently with this view, our questionnaire data revealed the best patient satisfaction resulted from mind-body therapies such as dietary interventions, relaxation, and psychotherapy.

Diet might be important in FM management, since macro- and micro-nutrients are known to affect oxidative stress, inflammation, and neuromodulation. Several food supplements (vitamins, probiotics, creatine, coenzyme Q10, and others) have been studied in relation to FM symptoms, but the results are inconclusive, except for a beneficial effect of vitamin D supplementation, given that FM patients generally present low Vitamin D levels [49,50]. However, plant-based and low-calorie diets have been shown to improve pain symptoms, sleep quality, and depression [51], by positively acting on the microbiota-gut-brain axis, even though an FM microbiota signature has not been identified yet [52], and ameliorating obesity, which shows some correlation with FM [53]. In our sample, the prevalence of obesity (20%) did not differ from that of the whole Italian population [54], but visceral adiposity, increased waist circumference, and the associated inflammation are common in non-obese, middle-aged, peri-menopausal women showing maximal FM prevalence [55]. Therefore, body-weight control should be a primary goal of FM patients, and this objective must also be achieved through adequate physical activity. The latest EULAR recommendations on FM management stress the importance of this issue giving the only ‘strong’ recommendation in favor of exercise [37]. Patients should be educated and encouraged to pursue behaviors that are functional to the self-management of a chronic disease [56]. Undoubtedly, it may be counterintuitive and scary for patients to start physical training, but they should become aware of the literature data confirming that a combination of aerobic and strengthening exercises can improve their pain and physical function [57,58].

Relaxation techniques, including among others deep breathing, progressive muscle relaxation, autogenic training, guided imagery (or visualization), biofeedback, mindfulness meditation, yoga, and tai chi [59], are aimed at counteracting stress and inducing a relaxation response, with slower breathing, lower blood pressure, and a reduced heart rate. Slow breathing is associated with enhanced parasympathetic activity, increased alpha, and decreased theta EEG waves [60], increased activity in prefrontal, motor, and parietal cortices, as well as in subcortical areas like the pons, thalamus, sub-parabrachial nucleus, periaqueductal gray, and hypothalamus [61]. These effects might be the reason why patients report positive effects on their wellbeing after relaxation techniques are implemented. Psychological/behavioral correlates to these changes lead to emotional control and psychological well-being in healthy subjects [62]. Moreover, enhanced vagus-mediated cholinergic signaling promotes immune and anti-inflammatory responses via the inflammatory reflex [63]. Accordingly, relaxation techniques can induce a downregulation of NF-κB-targeted genes, suggesting a beneficial effect in inflammation- and stress-related disease [64]. Although systematic reviews did not reveal strong correlations between relaxation techniques and FM improvement [65], different studies have reported positive effects on sleep, fatigue, depression, and anxiety [47], which generally worsen the experience of FM pain.

Thanks to the advances in the neurophysiology of pain, the latest edition of the Diagnostic and Statistical Manual of Mental Disorders (DSM-5) no longer includes pain as a specific mental disorder [66]. However, the PNEI paradigm helps to remember that the separation between psychological and physical pathologies is not possible, given the plethora of evidence regarding body-mind interconnections [27,28,67]. Accordingly, cognitive-behavioral therapy is the most widely studied and practiced psychotherapy for FM, showing improvements in pain, physical functioning, and mood [68]. In our work, personality, behavioral, and emotional styles were assessed using the CBA-H and SREIS questionnaires. Overall, the participants of our study were characterized by state anxiety, a depressive mood, and emotional instability, while their high capacity to perceive emotional activation was not sustained by the ability to understand and manage emotions. These features might reveal a low cortical activation for mentalizing capacity, and therefore, a balanced psycho-therapeutical approach focused on the training of their mentalizing skills could be advisable.

Psychological alterations in FM patients might worsen into full-blown psychiatric disorders, of which the most prevalent are anxiety disorders and depression [69]. Particularly, evidence indicates that childhood traumatic experiences might play a critical role in FM development and may be related to psychiatric comorbidities [70]. Therefore, various kinds of evidence indicate that psychological aspects are relevant for the management of FM patients [71]. Accordingly, results from our survey showed that the psychological characteristics of FM patients are correlated not only with the type of pain analyzed by the PD-Q test, but also with the perceived treatment effectiveness. Even if non-pharmacological therapies are considered altogether more effective, some people with social anxiety and interpersonal difficulties might feel uncomfortable with a mind-body approach, where the relationship with the therapist is very close. These observations suggest that treatments should consider tailored therapeutic strategies should be considered, based on the individual characteristics of FM patients, who therefore need an accurate anamnesis and a complete evaluation of their medical, social, and psychological history. In addition to pain being the main patient-reported symptom, other sensory impairments, as well as cognitive and emotional alterations, should be considered in order to choose the best therapeutic strategy, encompassing non-pharmacological approaches, on an individual basis, to pursue a better quality of life for patients.

### 4.4. Limitations of the Study

We are aware of some limitations to this study, which are linked to the intrinsic nature of online anonymous surveys, where the self-selection of participants (more prone to/capable of/interested in responding) cannot be avoided. FM patients received an invitation letter to participate in the study, but the sharing of the link through social networks could not be controlled, possibly involving some non-probability snowball sampling effects. We had to trust the patients on their self-reported conditions, but the findings that main FM features, such as gender bias, the prevalence of symptoms, comorbidities, and diagnostic delay, are significantly represented in our study population makes us confident in the validity of our data.

## 5. Conclusions

Our study showed:

− a prevalence of neuropathic/nociplastic pain in FM patients, correlated with anxiety, low mood, psychophysical discomfort, and an inability to relax;
− a perceived higher effectiveness of mind-body non-pharmacological treatments with respect to pharmacological ones;
− the role of pain types and psychological traits in determining the self-perceived effectiveness of therapies;
− a high self-perceived effectiveness of dietary interventions, relaxation techniques, and psychotherapy.

The data agree with the hypothesis of a central origin and development of FM, with a direct involvement of psychic functions controlling mood, emotions, and anxiety, suggesting the need for patient-tailored, integrated interventions for better therapeutic outcomes.

## Figures and Tables

**Table 1:** Percent frequencies of demographics in the sample of participants (n = 352).

Gender	Female	88.1
	Male	4.0
	No answer	7.9
Education	Primary	1.4
	Lower secondary	17
	Upper secondary	55.7
	Academic degree	18.5
	PhD or equivalent	7.1
	No answer	0.3
Marital status	Single	22.2
	Married/cohabitant	59.9
	Separated/divorced	15.6
	Widowed	2.3
Number of sons	0	37.2
	1	28.4
	2	25.9
	3	6.8
	>3	1.7
Employment	Grey-collar	39.8
	White-collar	11.9
	Blue-collar	8.8
	Shopkeeper	3.4
	Unemployed	35
	No answer	1.1

**Table 2:** Clinical characteristics of participants (n = 352).

	Min	Q1	Median	Mean ± s.d.	Q3	Max
Height (cm)	147	160	163	164 ± 6	168	193
Weight (Kg)	39	57	65	67.9 ± 15.4	76	125
BMI	15.6	21.3	24.2	25.3 ± 5.4	28.3	45.9
Patient age (years)	18	41	50	47.9 ± 10.8	56	86
Age-of-onset (years)	13	36	44	42.3 ± 10.1	50	83
Disease duration (years)	<1	5	9	11.7 ± 9.3	15	49
Diagnostic delay (years)	<1	1	3	6.35 ± 6	8	48

## Data Availability

The datasets used and analyzed during the current study are available from the corresponding author on reasonable request.
